# Epstein–Barr virus–driven molecular pathogenesis of primary pulmonary lymphoepithelial carcinoma

**DOI:** 10.3389/fonc.2025.1630415

**Published:** 2025-08-13

**Authors:** Mingyuan Xie, Dong Yao, Liping Lei, Chengjiang Tang, Biwen Mo

**Affiliations:** Department of Respiratory and Critical Care Medicine, the Second Affiliated Hospital of Guilin Medical University, Guilin, Guangxi, China

**Keywords:** pulmonary lymphoepithelial carcinoma, Epstein-Barr virus, genomic landscape, tumor immune microenvironment, immunotherapy

## Abstract

Primary Pulmonary Lymphoepithelial Carcinoma (PLEC) is a rare subtype of non-small cell lung cancer (NSCLC) that exhibits a strong association with Epstein–Barr virus (EBV) infection and shows distinctive geographic and ethnic predilections. Over the past decades, significant efforts have been made to elucidate the pathogenic mechanisms of PLEC, and progress in diagnosis, treatment, and disease monitoring has been achieved. This review focuses on EBV-driven oncogenic mechanisms in PLEC and explores the relationship between EBV infection, tumor progression, and clinical prognosis. We further summarize the molecular pathology, tumor immune microenvironment, and clinicopathological characteristics of PLEC. These insights may offer a theoretical foundation for EBV-targeted and immunotherapeutic strategies in PLEC.

## 1 Introduction

Lung cancer remains the second most commonly diagnosed malignancy and the leading cause of cancer-related deaths worldwide, accounting for an estimation of 2.2 million new cases and 1.8 million deaths annually (1–4). The majority (approximately 85%) of cases of lung cancers are classified as NSCLC according to the 2015 World Health Organization (WHO) classification of lung tumors (5). PLEC is a rare lung cancer subtype in NSCLC, characterized by undifferentiated carcinoma cells, ultrastructural features reminiscent of squamous cell carcinoma and abundant lymphoid stroma (6–9). The disease was first reported by Louis R. Berger and colleagues in 1987. Its prevalence shows a marked geographic bias and pertains to sex and age. Most of the cases were reported in Asia (mainly in Hong Kong and Guangdong, China), where young non-smokers were primarily affected, with female patients being more than male patients and concentrated ages between 51 and 55 years (10–15). The median age of onset of European patients is greater than that of the patients from Asia. The median age at diagnosis was 65 years (range: 15 years to 86 years) for European patients, in whom male patients and Caucasian patients accounting for 58.1% and 64.4%, respectively. Hence, the epidemiological characteristics of European patients with PLEC may be different from that of patients from Asia (16). In addition, over 90% of cases of PLEC from Asian are related to EBV infection. However, in the European and American population, the association of PLEC with EBV is low (8, 9). In the 2015 WHO classification, PLEC was categorized as other NSCLC or undifferentiated carcinoma, accounting for less than 1% of all NSCLC cases. In the 2021 WHO classification, PLEC has been reclassified as squamous cell carcinoma. Although PLEC has a more favorable prognosis compared to lung adenocarcinoma, squamous cell carcinoma, and large cell lung cancer (16–18), the five-year survival rate for PLEC remains around 74%, and effective improvement remains challenging (19, 20). This is attributed to a lack of diagnosis and treatment guidelines, clinical trials, and experience in targeted therapy and immunotherapy for PLEC. Therefore, we review the roles of EBV infection, molecular pathological changes, and immune features in PLEC, providing valuable insights for clinical research on targeted therapy and immunotherapy.

## 2 The pathogenicity of EBV in PLEC

### 2.1 EBV infection

EBV is among the most prevalent and persistent infections in humans. Approximately 95% of the world’s population experiences persistent, asymptomatic EBV infection throughout their lifetime, primarily affecting lymphocytes and oropharyngeal epithelial cells (21). There is evidence that the respiratory tract serves as the primary site of EBV hosting. The detection of EBV in bronchoalveolar fluid further suggests that lung tissue may serve as a primary reservoir for EBV (22). The life cycle of EBV encompasses two distinct phases: the latency period and the lytic phase (alternatively described as cleavage followed by reactivation). Following the initial infection which is typically asymptomatic, EBV establishes a lifelong persistent infection within the host. During latency, the EBV genome is replicated as an episome in the S phase of the cell cycle. Subsequently, these replicated episomes are distributed to daughter cells during cell division. The latent EBV expresses gene products that may possess carcinogenic properties, including Epstein-Barr virus nuclear antigen 1 (EBNA1), latent membrane protein-1 (LMP1) and microRNAs (miRNAs) (23–25). Notably, these gene products exhibit high expression levels in PLEC (15), indicating that the latent infection of EBV plays a significant role in the initiation and progression of PLEC. Osorio et al. addressed the epidemiological and experimental evidence of a potential role of EBV (26). However, the precise carcinogenic mechanisms underlying these associations remain to be elucidated through further investigation.

While EBV positivity has been considered a defining feature of PLEC, emerging studies have reported rare cases of EBV-negative PLEC. These EBV-negative cases challenge the current understanding of PLEC pathogenesis and suggest the existence of alternative oncogenic pathways. Studies from independent cohorts yielded similar results: patients with high EBV DNA before pretreatment or positive EBV DNA after treatment had significantly poorer progress free survival (PFS). Circulating EBV DNA levels provide prognostic value for survival and treatment response in patients with PLEC (27). Hence, elucidating the precise role of EBV in PLEC development is essential not only for understanding its typical viral-driven mechanism but also for distinguishing it from phenotypically similar but etiologically distinct tumors.

### 2.2 EBV-encoded gene products in PLEC

The role of EBV in tumorigenesis correlates with the expression levels of the latent genes and the extent of genomic aberrations in the host (28). EBV contributes to PLEC pathogenesis through the expression of multiple gene products, including latent proteins, non-coding RNAs, and virus-encoded miRNAs. In PLEC, EBV is primarily found in a latency type II infection pattern, with consistent expression of key latent genes such as LMP1, latent membrane protein 2A (LMP2A), and EBNA1, whereas EBNA2 is usually absent (29). In addition, EBV-encoded small RNAs (EBERs) and miRNAs are upregulated in tumor tissues. These products cooperatively regulate host signaling, immune evasion, and epigenetic modifications (30). The key viral gene products and their functional classifications are summarized in **Table 1**.

**Table 1:** Functional classification of EBV-encoded gene products in PLEC.

Gene product	Type	Function in PLEC	References
EBNA1	Latent protein	Maintaining episomal replication; regulating host gene expression; inducing local demethylation	(29, 31)
LMP1	Oncoprotein	Activating NF-κB, JAK/STAT, MAPK pathways; promoting transformation; upregulated in PLEC	(24, 29, 31)
LMP2A	Oncoprotein	Mimicing B-cell receptor signaling; enhancing cell survival; regulating NF-κB and indirectly LMP1	(29, 32)
EBERs	Non-coding RNA	Inducing interferon resistance; regulating host immune responses; upregulated in PLEC	(30, 33–36)
BART miRNAs	Viral miRNAs	Inhibiting apoptosis; suppressing p53 and PD-L1; modulating immune recognition and oncogenic signaling pathways	(30, 37, 38)

LMP1 is a constitutively active oncoprotein that mimics a tumor necrosis factor receptor (24). LMP1 plays a crucial role in the pathogenesis of EBV-associated tumors, such as nasopharyngeal carcinoma (NPC) and Hodgkin lymphoma (39, 40). Multiple studies have consistently reported that LMP1 expression is upregulated in PLEC tissues (15, 29, 41), reinforcing its oncogenic potential in a ‘pulmonary’ context. Mechanistically, LMP1 activates the tumor necrosis factor receptor-associated factor (TRAF)-mediated Nuclear Factor kappa-light-chain-enhancer of activated B cells (NF-κB) signaling pathway (42, 43). In line with this, Hong et al. observed frequent deletions of TRAF3 and NFKBIA in PLEC samples, suggesting that disruption of the LMP1–TRAF3 axis may represent a key molecular mechanism driving PLEC tumorigenesis (44). Unlike in NPC, where LMP1 activates both canonical and non-canonical NF-κB pathways primarily via TRAF2, recent findings suggest that in PLEC, TRAF3 downregulation may drive more sustained non-canonical NF-κB signaling, which may contribute to a more immunosuppressive tumor environment and resistance to apoptosis in PLEC.

LMP2A has been identified as the typical latency type II transcript encoded by EBV, and it is expressed in PLEC (29). Preliminary genetic studies suggested that LMP2A was not essential for *in vitro* growth transformation of B cells (32). However, recent studies have demonstrated that LMP2A can function as a simulated receptor, thereby promoting the malignant transformation of B cells. Nevertheless, the precise oncogenic mechanism of LMP2A remains to be elucidated. Studies have shown that LMP2A can indirectly regulate the expression of LMP1 by modulating the activity of NF-kB in epithelial cells (45). Consequently, the interaction of LMP2A with LMP1 in PLEC may enhance its oncogenic potential.

EBNA1 is a pivotal factor in the establishment of latent infection of EBV in proliferating B cells. EBNA1 has been demonstrated to stimulate replication of DNA, regulate transcription of both virus and host genes, and tether the virus to cell chromosomes (31). It has been demonstrated that EBNA1 exhibits a high degree of affinity for the recognition site in the human genome. The binding of EBNA1 to its target sequence has been shown to cause local demethylation, thereby promoting the activation of silent cell promoters (46). In a study by Wu et al., the expression of EBNA1 was found to be up-regulated based on genome sequencing of EBV isolated from 78 PLEC patients and 37 healthy controls (29). Despite the established role of EBNA1 as an oncoprotein expressed in all EBV-associated tumors (47), further investigation is required to elucidate its potential carcinogenic effect in PLEC.

Non-coding RNAs are transcripts that are not translated into proteins. EBER1 and EBER2 are EBERs that possess high abundance (typically reaching up to 10^7^ copies per cell) in latent infection (33). Their expression is considered the gold standard for identifying EBV-positive tumors via *in situ* hybridization (34). Chen et al. examined tumor tissues from 42 patients with PLEC by EBER detection, and 78.6% (33/42) showed positive results (48). Functionally, EBERs contribute to immune modulation and may promote cell survival. Recent findings indicate that EBER2 enhances B-cell growth by upregulating the deubiquitinase UCHL1, a process that may also be relevant in EBV-positive epithelial tumors (36).

EBV encodes two major clusters of miRNAs: miR-BHRF1 and miR-BART. The BamHI A rightward transcript (BART) cluster is preferentially expressed in epithelial tumors such as PLEC and NPC (37). Chen et al. performed RNA sequencing on fresh frozen tissues from eight patients with PLEC, and found that BART5-3P and BART20–3 were upregulated in PLEC, but not BART. The region between BART5-3P and BART20-3P is the dominant integration site of EBV. Functionally, miR-BART5-3p suppresses TP53-mediated apoptosis by directly targeting the 3′UTR of p53 mRNA, while miR-BART20-3p downregulates MICB, an NKG2D ligand, thereby reducing NK cell-mediated tumor surveillance. Of these eight patients, five had low expression of p53 and programmed death-ligand 1(PD-L1), and the prognosis was poor (30). It has been reported that the high expression of miR-BART in NPC promotes cancer development by targeting various cell and viral genes. Although primarily studied in NPC, the upregulation of miR-BART in PLEC suggests shared mechanisms of EBV-driven tumorigenesis (38).

### 2.3 EBV integration sites contribute to the initiation of PLEC

Chen et al. identified 288 integration breakpoints of EBV on chromosomes after whole exome sequencing in 128 PLEC patients. They found that the intergenic regions were the preferred integration sites of EBV in PLEC. EBV was easily integrated into the intergenic and intronic regions of two up-regulated miR-BARTs, namely BART5-3P and BART20-3P. These fragile regions were susceptible to DNA damage, which increases the possibility of EBV DNA insertion into the host genome and contributes to the occurrence of PLEC (30). Wu et al. identified 179 EBV host integration sites by bioinformatics methods, only 7 sites were validated by targeted PCR amplification and Sanger sequencing (29). These validated integration events occurred in three patients in advanced stages of PLEC (stages III and IV), suggesting to us that EBV integration may be somehow associated with the stage of tumor progression. The investigators identified an integration hotspot on chromosome 4q28.3 subband, a finding that is particularly striking because it may point to a key oncogenic mechanism. The hotspot was integrated twice in the same patient, further highlighting its potential importance in tumourigenesis. In addition, another integration breakpoint was located in the adjacent subband 4q31.21, which further narrowed the scope of the study and allowed scientists to more precisely investigate the effects of these integration events on gene expression and function (29). In summary, the above studies not only revealed the integration sites of EBV in host cells, but also preliminarily explored the effects of these integration events on gene expression and function. These findings provide new clues for a comprehensive insight of the mechanism of EBV-associated tumourigenesis, as well as potential targets for future therapeutic strategies.

### 2.4 Immune deficiency and EBV infection

Although the immune system can largely control EBV infection, the virus cannot be eliminated. To produce new viral progeny, EBV is reactivated from the latently infected cells. After analyzing the copy number variations of 46 cases of PLEC, Hong et al. found that the narrow region of 9p21.3 (chr9: 22028316-22041442) showed a focal and significant deletion, and the nearby region within 9p21.3 also showed a high-frequency deletion involving the type I interferon (IFN) gene. Type I IFN is a frontline defense against viral infection and a key component of host-virus confrontation. The level of CD8^+^ tumor-infiltrating lymphocytes (TILs) in tumors with the 9p21.3 deletion was lower than that in tumors without the 9p21.3 deletion. This suggests that frequent loss of type I IFN gene may lead to a lack of host immune response to the virus and persistent EBV infection in PLEC (44). These findings suggest that deletions in immune-regulatory loci, such as type I IFN genes on 9p21.3, may contribute to persistent EBV infection and immune evasion in PLEC (49).

### 2.5 Epigenetic susceptibility of the host genome in PLEC

EBV-encoded oncoproteins regulate cellular epigenetic mechanisms to reprogram viral and host epigenetic genomes, particularly in the early stages of infection (50). Abnormal epigenetic modifications mainly include CpG methylation and histone modification. In epithelial cells, LMP1 can upregulate DNA methyltransferase (46). Although LMP1 is highly expressed in PLEC (15, 41, 44), its carcinogenic effect through the regulation of epigenetic mechanisms needs further investigation.

### 2.6 A proposed model for EBV-driven PLEC

**Figure 1:**
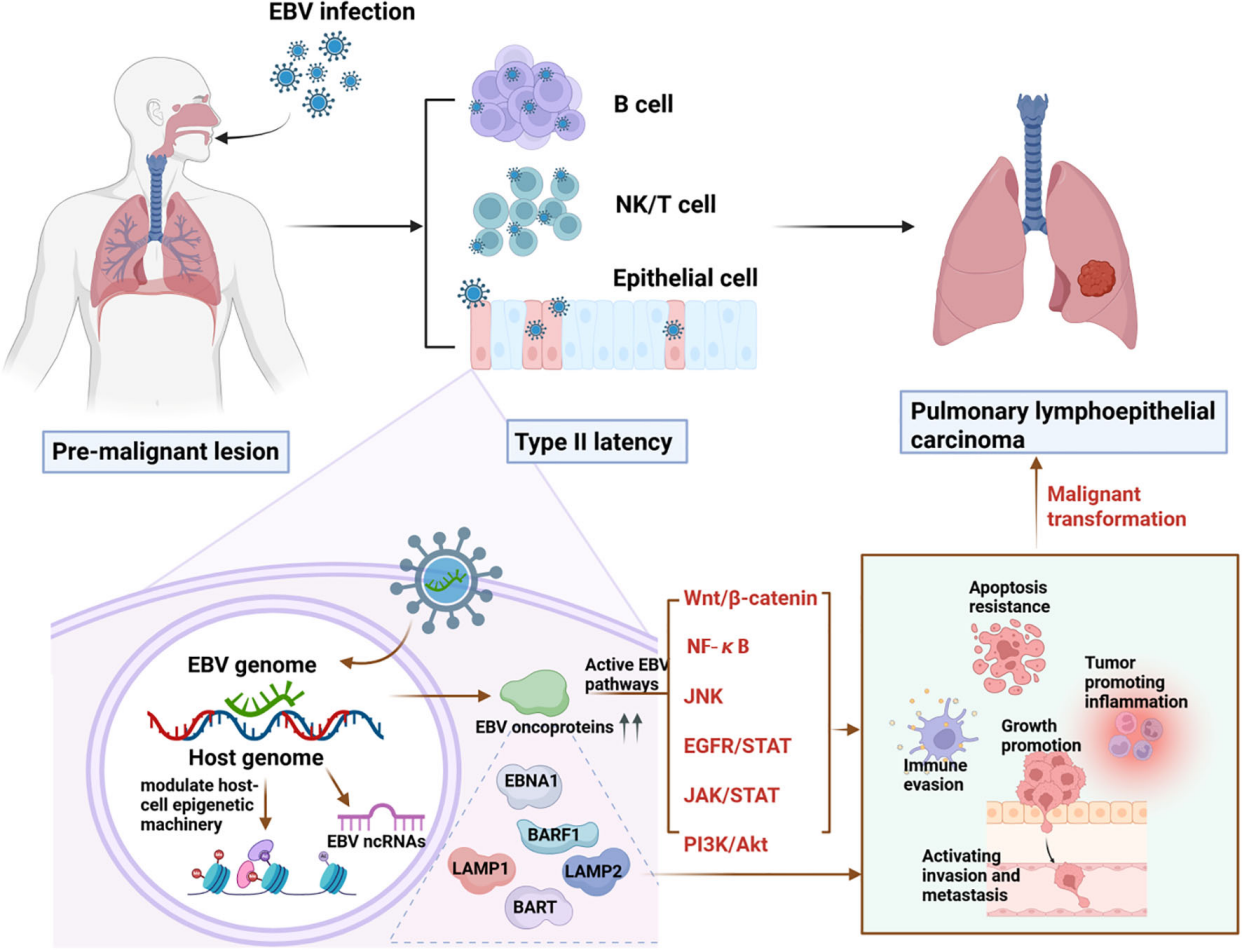
A proposed mechanism for EBV infection-driven PLEC.

EBV contributes to PLEC tumorigenesis through a sequential cascade involving epigenetic reprogramming, oncogenic signaling activation, and dysregulation of host gene expression. Thus, we propose a model for EBV-driven PLEC as shown in **Figure 1**. Initially, EBV infection alters epigenetic marks including DNA methylation and histone modifications in bronchial or alveolar epithelial cells, thereby disrupting normal gene expression to promote uncontrolled proliferation, and impair apoptosis. These epigenetic changes facilitate the activation of multiple oncogenic pathways, such as NF-κB, PI3K/AKT, and JAK/STAT, mediated by viral proteins like LMP1 and LMP2A. Furthermore, EBV-encoded gene products—including EBNA1, BARF1, and BART miRNAs—enhance tumor progression by fostering immune evasion, chronic inflammation, and resistance to apoptosis. Together, these mechanisms form an integrated network driving the initiation and advancement of PLEC.

## 3 Association of EBV with the progression and prognosis of PLEC

Serum EBV DNA not only holds significant value in diagnosis, but also provide valuable information for disease monitoring. Ngan et al. detected free EBV DNA in the serum of PLEC patients using quantitative real-time polymerase chain reaction (q-PCR) and found that the level of serum EBV DNA in patients with primary PLEC usually changes after treatment (51). A rapid decline in EBV DNA in the serum may be associated with a favorable response to treatment, while the rising level of EBV DNA after treatment may indicate drug resistance or recurrence of the tumor. Xie et al. determined the EBV DNA titer in 429 PLEC patients, further confirming the feasibility of monitoring treatment response in late-stage cases (52). They showed that serum EBV-DNA can reflect tumor burden and can be used as a tumor marker to assess treatment response. By tracking the EBV DNA levels before and after treatment, researchers can determine the duration of an effective treatment for PLEC patients.

EBV DNA positivity, particularly values exceeding 10,000 copies/mL, has been associated with poorer survival outcomes in PLEC. Several studies have demonstrated that cancer staging in patients with PLEC is closely correlated with changes in EBV DNA copy number. The results of a recent study showed that the median value of EBV DNA was as high as 29,500 copies/mL (ranging from 0 to 28,400,000 copies/mL), a value that is much higher than those of previous studies (13). This may be related to the fact that most of the PLEC patients selected in the study were in advanced stages. The study also found a significant association between high EBV DNA subgroups (i.e., ≥41,900 copies/mL) at baseline and poorer survival prognosis (13). Hence, EBV DNA may become an important biomarker for predicting patient prognosis.

## 4 Genetic landscape of PLEC

### 4.1 Copy number variations and altered signaling pathways in PLEC

Numerous copy number variations (CNVs) have been identified in PLEC, and their biological and clinical impacts are summarized in **Table 2**. The CNVs, characterized by frequent gains in oncogenic regions and losses in tumor suppressor loci, play a crucial role in shaping the molecular landscape and immunogenicity of PLEC.

**Table 2:** Copy number variations (CNVs) reported In PLEC.

Chromosome position	Copy number change	Impact	References
1q(16%),5p(32%), 12p(54%) and 12q(48%)	Gain	Amplification of chromosome 12 results in activation of signaling pathways including MAPKs, JAK/STAT, and cell cycle.	(44, 59)
3P (49%)(16%), 5q (47%),9p(14%), 13q (36%), 14q (60%) (14%)and 16q (39%)	Loss	The loss of 14q and 16q led to the inactivation of a range of negative regulatory factors in the NF-kB signaling pathway, including TRAF3 [14q32.3, 80%], NFKBIA[14q13, 52%], NLRC5 [16q13, 52%], and CYLD [16q12.1, 48%].	(44, 59)
7p11.2, 9p24.1, 11q13.3 and 12p13.2	Amplification	Amplification in 11q13.3 contains the CCND1 gene, and amplification of CCND1 may drive cell cycle progression and promote tumorigenesis.	(44)
3p21.31, 3p25.3, 5q14.1, 9p21.3, 11q23.3, 13q14.2, 14q32.32	Deletion	Patients with 9p21.3 loss are significantly associated with poor survival.	(44)
3p24.1, 5q12.3, 11q24.3 and 14q11.2	Deletion	/	(30)
9p24.1 (Chromosome 9:5422115-5639664)	Amplification	The amplification region contains four protein coding genes, CDs 274 (encoding PD-L1), PLGRKT (encoding plasminogen receptor KT), RICl (encoding RIC1) and PDCDILG2 (encoding PD-L2). In these genes, CD274 and PDCDILG2 encode two ligands of the programmed death-1 receptor, PD-L1 and PD-L2, respectively.	(58)
3P, 5q, 13q and 16q	Loss	/	(42)
12q	Gain	/	(42)

Based on the mutation profile, key signaling pathways associated with PLEC have been suggested in PLEC, such as cell cycle, JAK/STAT and NF-κB pathways. Mutations or deletions of TP53, amplification of MDM2 and CCND1, deletions of CDKN2A/B and RB1 are implicated in the dysregulation of cell cycle in PLEC (42, 44, 53, 54). The frequent dysregulation of the JAK/STAT pathway in PLEC is primarily due to the deletion of CISH, followed by mutations or deletions of PTPRD, and mutations or amplification of JAK2 (44). CISH encodes a cytokine-induced SH2-containing protein from the SOCS family, which serves as a key negative regulator of the JAK/STAT pathway (55). PTPRD encodes a tumor suppressor that negatively regulates JAK/STAT pathway by dephosphorylation and inactivation of STAT3 (56). In addition to deletions of key negative regulators of NF-κB signaling pathway (including TRAF3, CYLD, NFKBIA, and NLRC5), somatic mutations or amplification of several components of the canonical NF-κB pathway, including FADD, TRAF2, TRAF6 and CARD11, have been identified in PLEC. FADD is an apoptosis adaptor protein that activates the NF-κB signaling pathway through recruitment of caspase-8. TRAF2 and TRAF6 are members of the TRAF protein family that mediate NF-κB signaling pathway activation and participate in the regulation of inflammation, antiviral response and apoptosis (57).

Interestingly, studies have identified mutual exclusivity between LMP1 overexpression and the three key pathway (cell cycle, JAK/STAT and NF-κB) aberrations (44). Furthermore, several regulatory genes involved in immune escape, such as CD274 (PD-L1) and PDCD1LG2 (PD-L2), were found to be amplified in PLEC (44, 58). These findings suggest that both somatic alterations and viral factors may collaborate in the tumorigenesis and progression of PLEC.

### 4.2 Drive mutations in PLEC

Studies have shown that approximately 73.9% of Chinese patients with NSCLC harbor at least one actionable mutation, as defined by the National Comprehensive Cancer Network guidelines, including mutations in the epidermal growth factor receptor (EGFR), anaplastic lymphoma kinase (ALK), Kirsten rat sarcoma viral oncogene (KRAS), v-raf murine sarcoma viral oncogene homolog B1 (BRAF), and repressor of silencing 1 (ROS1) (60). EGFR mutations, including point mutations and small insertions/deletions, were the first identified pharmaceutically targetable mutations in NSCLC and remain the most widely used predictive biomarker for EGFR tyrosine kinase inhibitors. The most common EGFR mutations associated with sensitivity to tyrosine kinase inhibitors include the deletion of exon 19 (approximately 45% of patients with EGFR mutations) and the L858R mutation in exon 21 (approximately 40%) in NSCLC (61). In PLEC, the identified EGFR mutations include the L858R mutation in exon 21, non-L858R mutation in exon 21, deletions in exon 19, mutations in exon 20, and mutations in exon 18 (**Table 3**) (54, 62–64). Overall, alterations in EGFR and ALK genes are relatively rare in primary PLEC However, there may still be some special cases with EGFR or ALK gene alterations that interact with other factors or are related to individual differences of patients, tumor heterogeneity, or detective methods (65). The current data tentatively suggests that EGFR-targeted therapy is not suitable for patients with advanced PLEC due to lack of the typical driver mutations as observed in NSCLC. The low prevalence of classic driver mutations in PLEC implies that PLEC may be driven by a distinct tumorigenic pathway, rather than by conventional oncogenic mutations found in NSCLC.

**Table 3:** Driving gene mutations in PLEC.

Gene	Mutation position	Detection method	Detection rate	References
EGFR	21 exon L858L	PCR amplification combined with direct sequencing	2.7%(1/36)	(62)
EGFR	21 exon L858L	TaqMan real-time PCR amplification	2.4%(1/42)	(63)
EGFR	21 exon non- L858L site	PCR amplification combined with direct sequencing	8.7% (4/46), 6.1% (4/66)	(54, 64)
EGFR	20 exon	PCR amplification combined with direct sequencing	6.7% (3/46), 4.5% (3/66)	(54, 64)
EGFR	19 exon deletion	PCR amplification combined with direct sequencing	2.2% (1/46), 1.5% (1/66)	(54, 64)
EGFR	18 exon	PCR amplification combined with direct sequencing	2.2%(1/46)	(54)
EGFR	/	Amplification of blocking mutation system PCR, TaqMan real-time PCR amplification, NGS	0 (0/11), 0 (0/7), 0 (0/30), 0 (0/32),0 (0/27)	(17, 20, 66–68)
ALK rearrangement	/	IHC	0(0/30)	(67)
ROS1 fusion status	/	FISH, NGS	0(0/30),0 (0/11), 0 (0/7), 0(0/27)	(17, 20, 66, 68)
BRAF	/	NGS	0 (0/11), 0 (0/7), 0(0/27)	(17, 20, 66)
KRAS	/	NGS	0 (0/11), 0 (0/7), 0(0/27)	(17, 20, 66)
EML4-ALK fusion	/	IHC,FISH	1/1	(65)

PCR, Polymerase Chain Reaction; IHC, Immunohistochemistry; FISH, Fluorescence in Situ Hybridization; NGS, next-generation sequencing.

## 5 Immune landscape of PLEC

### 5.1 Adaptive immune cells in PLEC microenvironment

**Figure 2:**
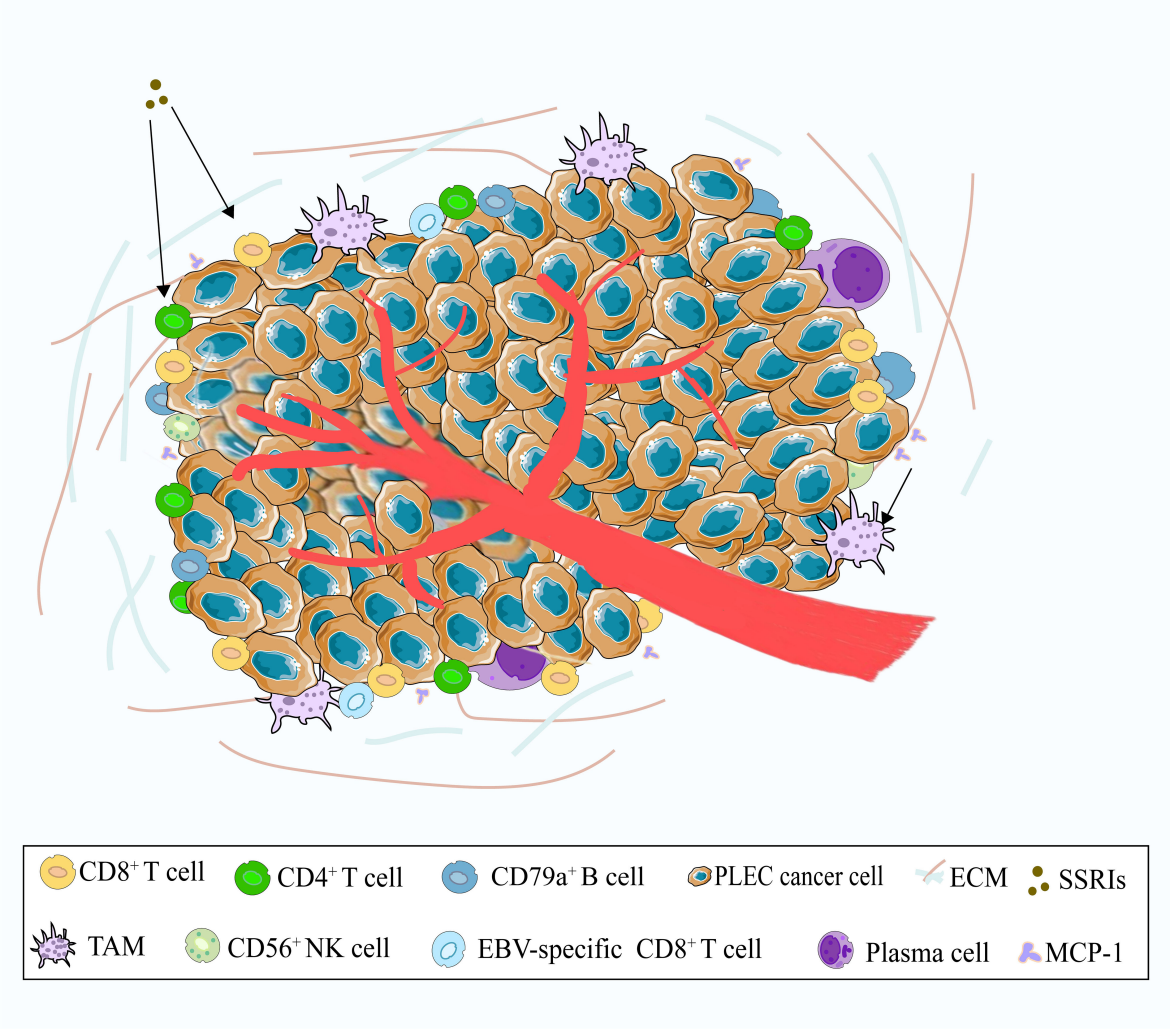
A schematic illustration of the tumor microenvironment of PLEC. The main immune cells include CD8^+^ T cells, CD4^+^ T cells, CD79a^+^ B cells, and TAMs. Other cells such as EBV-specific CD8^+^ T cells, CD56^+^ NK cells, and plasma cells are relatively rare. In addition, SSRIs may activate CD8^+^ T cells and CD4^+^ T cells, while MCP-1 may recruit TAMs.

The tumor microenvironment (TME) refers to the complex milieu surrounding a tumor, encompassing cellular components, extracellular matrix (ECM), and vascular networks, which collectively influence tumor initiation, growth, and metastasis (**Figure 2**). TME usually includes immune cells, stromal cells, ECM and other secreting molecules, blood and lymphovascular network. The immune cell population within the TME includes T cells, B cells, tumor-associated macrophages (TAMs), dendritic cells (DC), natural killer cells (NK), neutrophils and myeloid suppressor cells (MDSCs), and others. The various subsets of immune cells in the TME exhibit distinct functions, which influence tumor progression through multiple mechanisms (69). Research has found that the recurrence risk of stage I NSCLC may be attributed to alterations in the immune and metabolic microenvironment (70). PLEC is a malignant epithelial tumor characterized by pronounced lymphocytic infiltrates. Kasai et al. observed TILs in EBV-positive PLEC. Most CD3-positive T cells were labeled as CD8 and TIA-1 positive but were negative for granzyme-B, indicating that TILs are resting cytotoxic T-lymphocyte (CTLs) (71). Chang et al. analyzed the lymphocyte composition in the stroma surrounding PLEC tumor cells by immunohistochemistry, and they found that CD8^+^ cells and B cells were present in all cases. In total, the number of stained cells of CD8 positive cells exceeded that of B cells (72). The results of the study by Kobayashi et al. showed that in PLEC, infiltrated T cells were more than B cells, and CD8 positive cells were more than CD4 positive cells (73). Yo Kawaguchi reported a case about a 70-year-old male with PLEC, in which tumor regression occurred during treatment with selective serotonin reuptake inhibitors (SSRIs). Subsequently, histological examination revealed infiltration of CD3^+^, CD4^+^ and CD8^+^ lymphocytes around the tumor. It has been hypothesized that SSRIs may activate these lymphocytes, potentially leading to spontaneous tumors regression (74). There are also studies reporting the impact of neoadjuvant therapy on PD-L1 expression and CD8^+^ lymphocyte density in NSCLC (75).Studies have demonstrated that EBV-specific CD8^+^ TILs in EBV-driven PLEC exhibit heterogeneity and partial deficiency in PD-1 expression (76). Furthermore, other researchers have identified polyclonal plasma cells, polymorphonuclear granulocytes, and CD56^+^ NK cells, alongside CD3^+^ T-lymphocytes and CD79a^+^ B-lymphocytes among the infiltrating cells in PLEC (77, 78). To date, the relationship between lymphocyte infiltration and patient prognosis remains debated in PLEC. The distribution of various lymphatic subpopulations in PLEC requires further elucidation through single-cell sequencing and large multicenter studies (79).

### 5.2 TAMs in PLEC microenvironment

TAMs are among the tumor-infiltrating immune cells (TIIC) within tumor microenvironment. TAMs are derived from MDSCs or monocytes and play pivotal roles in promoting tumor growth, metastasis, angiogenesis, and immunosuppressive functions (80). PLEC is closely associated to a variety of mononuclear inflammatory cells, including a substantial population of TAMs, which contribute to its pathophysiology. The study of Wong et al. demonstrates that the expression of monocyte chemoattractant protein-1 (MCP-1) in PLEC tumor cells plays a critical role in the recruitment of TAMs in PLEC (81). Wang et al. show that pre-treatment monocyte-to-lymphocyte ratios (MLR) may serve as an independent prognostic marker in patients with PLEC and could guide the optimization of treatment strategies (82). TAMs in PLEC tumors are thought to contribute to the inhibition of anti-tumor immune response. However, given the limited sample sizes in previous studies, this hypothesis requires further validation in larger cohort studies.

### 5.3 Expression and clinical significance of PD-L1 in PLEC

The programmed cell death protein 1 (PD1) - PD-L1 axis presents a critical immune checkpoint pathway, which can be hijacked by cancer cells to evade immune surveillance (83, 84). PD-L1 expression is a biomarker for improving the survival of advanced PLEC and the potential effectiveness of immunotherapy (85). Blockade of PD1-PD-L1 axis in NSCLC has led to durable objective response and significantly improved survival compared to conventional therapies (86). The combination of PD-1/PD-L1 and lymphocyte activation gene 3 (LAG-3) is associated with the clinical activity of immune checkpoint inhibitors (ICIs) in metastatic PLEC (87). Studies have shown that PD-L1 expression in PLEC is elevated compared to the average levels observed in NSCLC, suggesting that immunotherapy may be a promising treatment strategy for PLEC (18, 64). However, the prognostic value of PD-L1 expression in PLEC tumor cells remains controversial (88, 89). The discrepancies in findings across studies may be attributable to several factors, including the types of PD-L1 antibodies used, varying thresholds for defining positive expression, and differences in specimen collection methods (**Table 4**). These differences highlight the urgent need for standardized PD-L1 assessment protocols in PLEC, Multi-center, large-sample clinical studies aiming to address the consistency of efficacy of different PD-L1 expression cut-offs and PD-L1 antibodies have become important for the immunotherapy of PLEC.

**Table 4:** A list of studies of PD-L1 expression in PLEC.

Cases No.	PD-L1 expression		Cut-off	Relationship with other immune biomarkers	Prognostic or predictive value	References
	TC (%)	IC(%)				
31	93.5%		1%	ND	ND	(18)
47	61.7%		1%	ND	associated with extended DFS	(30)
57	79%		1%	ND	ND	(42)
27	75%		1%	ND	PD-L1 expression have longer PFS and OS than those with negative PD-L1 expression	(62)
66	75.8%		5%	ND	ND	(64)
59	96.6%		1%	ND	ND	(66)
59	91.5%		5%	ND	ND	(66)
19	78.9%		1%	ND	ND	(88)
67	65.6%		5%	PD-L1 (+)in TCs and P53(+)	associated with longer DFS	(89)

PD-L1, programmed death ligand-1; TC, tumor cell; IC, immune cell; ND, not described; DFS, disease-free survival; PFS: progression-free survival; OS: overall survival.

### 5.4 Therapeutic prospects

**Figure 3:**
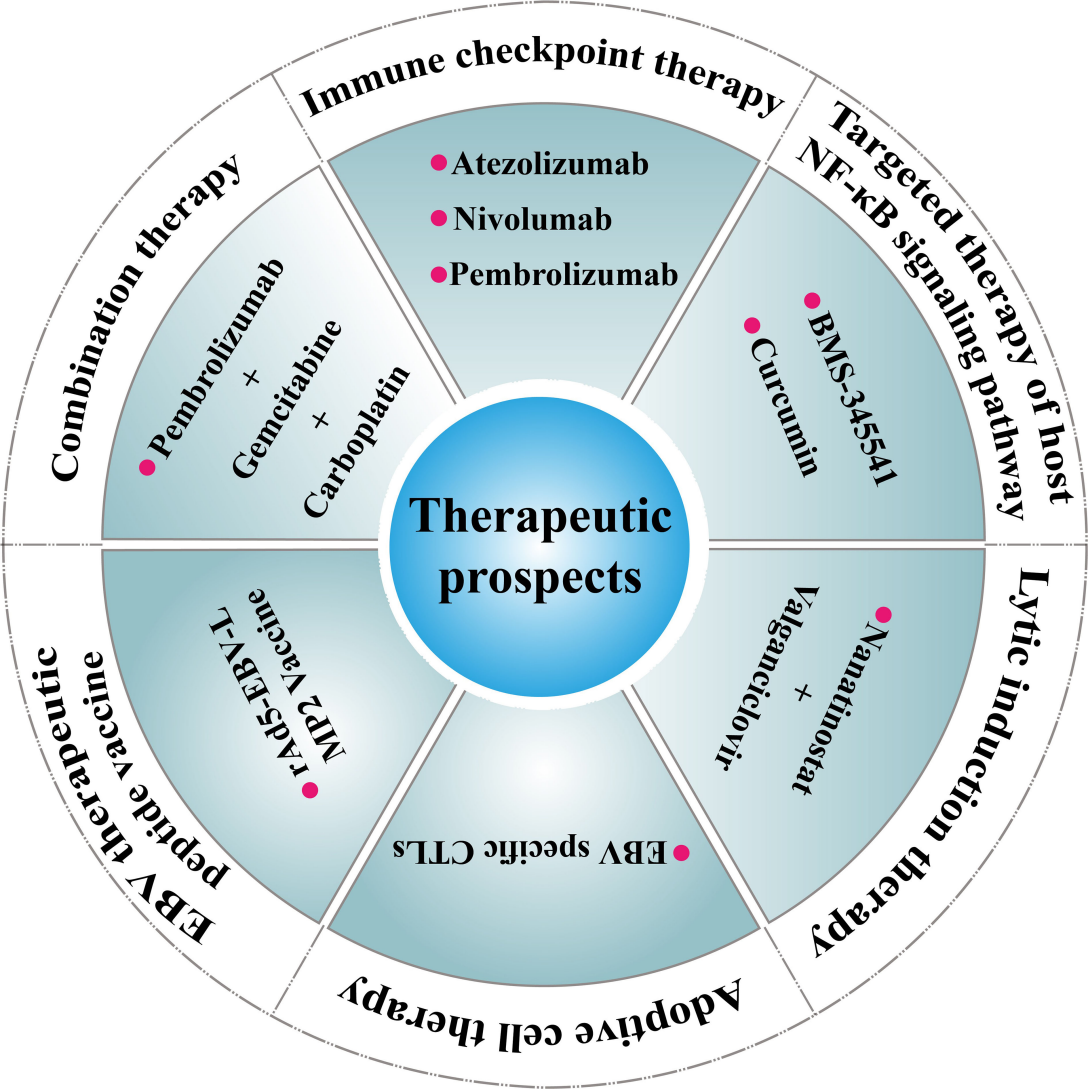
Therapeutic prospects for PLEC. Immune checkpoint therapy (e.g., Atezolizumab, Nivolumab, Pembrolizumab) Ref (90); Targeted therapy of host NF-κB signaling pathway (e.g., BMS-345541, Curcumin Ref) (93); Adoptive cell therapy (e.g., EBV specific CTLs Ref (96); Lytic induction therapy (e.g., Nanatinostat + Valganciclovir) Ref (97); EBV therapeutic peptide vaccine (e.g., rAd5-EBV-LMP2 Vaccine) Ref (98); Combination therapy (e.g., Pembrolizumab+Gemcitabine+Carboplatin) Ref (99).

Currently, there are no standardized treatment protocols for PLEC due to its rarity. However, its association with EBV infection, high PD-L1 expression, and activation of oncogenic signaling pathways such as NF-κB suggests several potential therapeutic strategies (**Figure 3**).

ICIs have shown promise in EBV-associated tumors and may offer clinical benefit in PLEC (90, 91). Case series and retrospective analyses have reported durable responses to PD-1/PD-L1 blockade in patients with advanced or relapsed disease. Nonetheless, variability in PD-L1 detection methods, cut-off thresholds, and tumor heterogeneity complicates patient selection. Refinement of biomarker assessment—including co-expression of cytotoxic T-lymphocyte-associated protein 4 (CTLA-4), Lymphocyte Activation Gene 3 (LAG3), or tumor mutational burden (TMB) status—may help identify responders more accurately (92).

Given that LMP1-mediated activation of the NF-κB pathway is a hallmark of EBV-driven oncogenesis, this axis represents another promising target. Preclinical studies in related EBV-positive malignancies have explored the use of NF-κB pathway inhibitors, such as proteasome inhibitors or natural compounds (93), though their clinical efficacy in PLEC remains untested (94, 95).

In addition, EBV-directed therapies are under development. Approaches such as lytic induction therapy combined with antiviral agents (e.g., ganciclovir), adoptive transfer of EBV-specific CTLs, and EBV peptide vaccines have shown encouraging results in NPC and post-transplant lymphoproliferative disorders (96–98). These modalities could be translated to PLEC with further validation (18, 95).

Combination therapies, such as ICIs plus chemotherapy, radiotherapy, or anti-angiogenic agents, may also enhance antitumor responses by modulating both tumor and immune compartments (99). Given the immunologically active microenvironment of PLEC, multimodal regimens may offer a rational approach, though prospective clinical trials are needed (92).

Briefly, these emerging strategies highlight the importance of tailoring treatment based on both viral and immune profiles in PLEC. Future research should focus on validating these approaches in clinical settings and establishing disease-specific therapeutic guidelines.

## 6 Histopathological diagnosis and immunohistochemical features of PLEC

**Figure 4:**
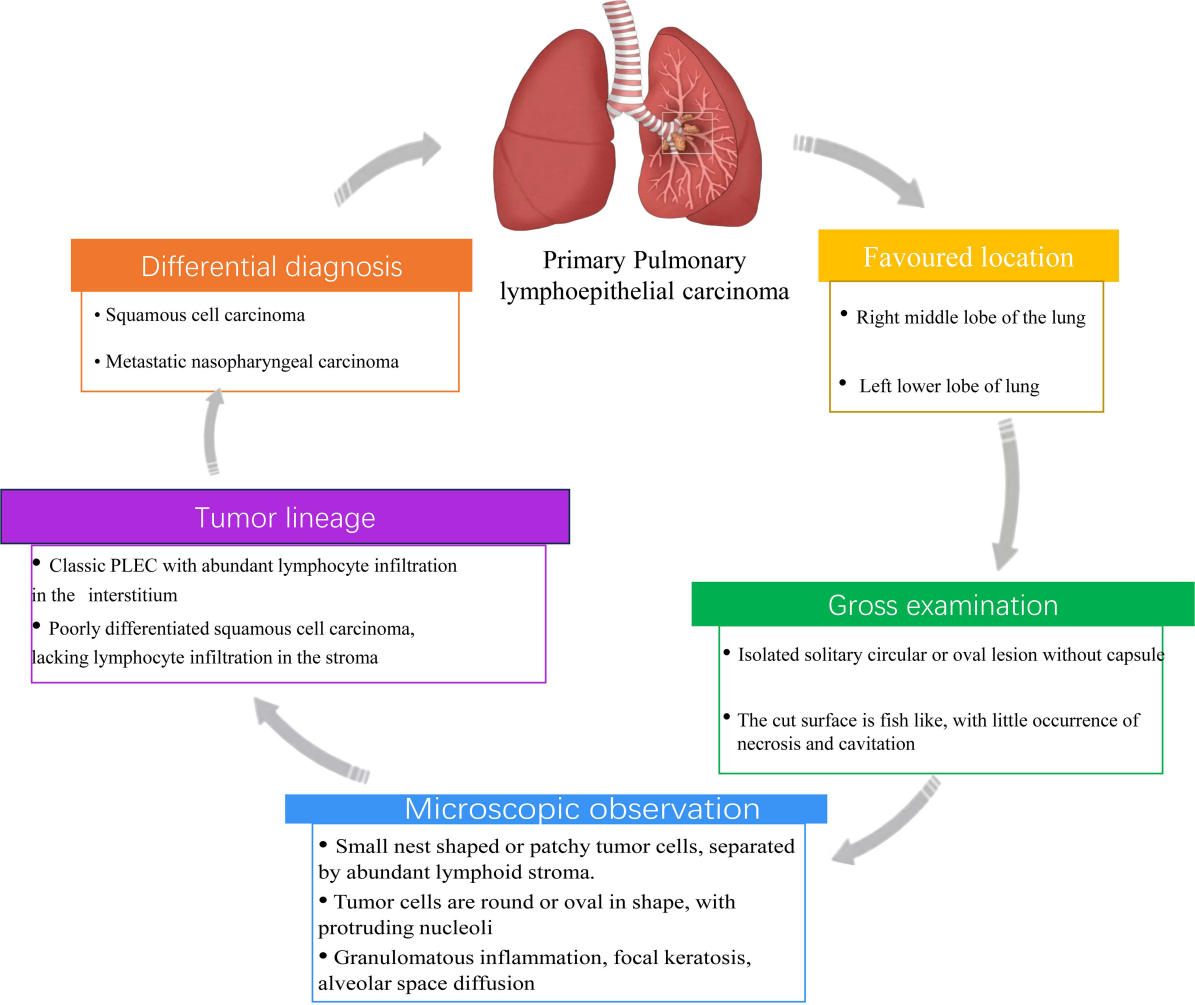
Histopathological and diagnostic characteristics of PLEC. This schematic summarizes key features of PLEC including favored anatomical locations, gross morphology, microscopic features, tumor lineage, and differential diagnosis.

Although the molecular and immunological features of PLEC have been well characterized, histopathological evaluation remains essential for accurate diagnosis, particularly in differentiating PLEC from poorly differentiated squamous cell carcinoma. This section summarizes the key histological and imaging features of PLEC, including its lymphoepithelioma-like morphology and immunohistochemical profile, which complement molecular findings (**Figure 4**). Studies have shown that PLEC primarily occurs in the right middle lobe and left lower lobe of the lung. This conclusion has been corroborated by retrospective studies, and further investigations conducted in Macao suggest that the distribution of PLEC in the lungs follows a specific pattern (100–102). PLEC typically presents as isolated, solitary, round or oval lesions, lacking a capsule and well-defined borders upon gross examination. The cross-sectional appearance is characterized by a pale white or brown color, with good elasticity and a fish-like shape, rarely exhibiting necrosis or cavitation (72, 103, 104). These features are helpful for the initial identification of PLEC during surgery. Microscopically, PLEC is characterized by small, nest-like or patchy hyperplasia of epithelial tumor cells, which were separated by abundant lymphoid stroma or lymphocyte aggregates. The tumor cell nuclei are large, round or oval with prominent nucleoli (11, 105–107). Although PLEC may be histologically confused with Undifferentiated Nasopharyngeal carcinoma (UNPC), it exhibits features such as granulomatous inflammation, focal keratosis, alveolar space diffusion and squamous differentiation pattern, which distinguish it from UNPC (79). In addition, PLEC and lung squamous cell carcinoma (LUSC) exhibit similar biomarkers expression patterns in immunohistochemical analysis, indicating that PLEC displays the characteristics of squamous cell differentiation. However, the abundant lymphocyte infiltration and large tumor cell nuclei in PLEC distinguish it morphologically from LUSC (48). Recent studies have identified a spectrum of morphological characteristics in PLEC. At one end of this spectrum, classic PLEC is characterized by stroma rich in lymphocyte infiltration, while at the other end, it resembles poorly differentiated squamous cell carcinoma with minimal interstitial lymphocyte infiltration. This spectrum change suggests that PLEC with absent interstitial lymphocyte infiltration is frequently misdiagnosed as squamous cell carcinoma in clinical practice (79). Therefore, comprehensive pathological and immunohistochemical analysis should be conducted for suspected PLEC cases to prevent misdiagnosis. The clinicopathological features, especially the spectrum of lymphoid infiltration and histological overlapping with squamous cell carcinoma, underscore the diagnostic challenge of PLEC and the importance of combined morphological and molecular evaluation.

The immunohistochemical pattern is characterized by reduced p53, deletion of c-erbB-2 and high expression of PD-L1. The combination of these features not only helps us understand its tumor biological mechanism, but also provides important clues for clinical treatment. The reduced expression of p53 may be related to the uncontrolled proliferation of tumor cells and the increased tolerance to DNA damage. Specifically, the absence of p53 enables tumor cells to evade normal cell cycle checkpoints and apoptotic procedures, further promoting the proliferation of tumor cells and the malignant transformation of tumors. The absence of c-erbB-2 is a common feature. This absence may indicate that the dependence of tumor cells on external growth signals is weakened, and the growth of tumors no longer depends on the excessive activation of c-erbB-2. The deletion of c-erbB-2 may also lead to tumor cells activating proliferation signals through other alternative mechanisms, thereby maintaining their proliferation and survival abilities. PLEC often shows high expression of PD-L1, which indicates that the tumor may adopt an immune escape strategy to avoid recognition and clearance by the host’s immune system. This immunohistochemical pattern provides us with a more comprehensive biological perspective of PLEC and important information for developing treatment strategies.

## 7 Unresolved questions and future challenges

Despite growing insights into the molecular and immune features of PLEC, several key questions remain unanswered. First, the prognostic significance of PD-L1 expression in PLEC is inconsistent. While some studies link PD-L1 positivity to favorable outcomes, others report no correlation (17, 108). These discrepancies may reflect differences in cut-off thresholds (e.g., 1% vs. 5%), antibody clones, detection platforms, and limited sample sizes. Standardized protocols and larger, prospective studies are needed to clarify its clinical relevance. Second, although EBV latency type II is shared with other EBV-driven tumors such as NPC and EBV-associated gastric cancer (EBVaGC), its downstream effects in PLEC remain poorly defined (18, 109, 110). In NPC, latency II activates LMP1/NF-κB and LMP2A/PI3K-Akt pathways, promoting immune escape and tumor progression. PLEC, however, appears to exhibit distinct immune infiltration patterns and unique EBV-encoded miRNAs expression profiles (e.g., BART5-3p, BART20-3p), possibly targeting lung-specific pathways such as interferon signaling (30). The tissue-specific consequences of latency II in the pulmonary context require further investigation. Third, the immune microenvironment of PLEC is still poorly characterized (109). Single-cell or spatial transcriptomic analyses have yet to be applied, limiting understanding of immune cell composition, heterogeneity, and interactions with EBV-infected cells. In addition, EBV strain diversity in lung tumors and its clinical impact remain unexplored. Finally, the lack of *in vivo* models or lung organoids for EBV infection hinders functional validation of proposed mechanisms (109). Addressing these gaps will require integrative genomic and immunologic studies, improved model systems, and cross-disciplinary collaboration. A deeper understanding of these unresolved issues is essential for advancing diagnosis and therapy in PLEC.

## 8 Conclusion and future perspectives

PLEC is a rare subtype of NSCLC, and its development and progression are influenced by genetic alterations, immune activity, and EBV infection. Although some progress has been made in understanding the molecular pathobiology of PLEC, large-scale clinical studies are still needed to clarify its molecular mechanisms, clinical characteristics, and to inform standardized treatment strategies. In the future, it will be interesting to compare the transcriptomic profiles between infected cells and adjacent non-infected cells using single-cell multi-omics technologies to identify virus-induced early carcinogenic events in PLEC. Additionally, patient-derived organoids from PLEC may be constructed, and used in combination with efficient gene-editing tools such as CRISPR-associated protein 9 (CRISPR-Cas9) to evaluate the impact of genetic biomarkers on drug response. This approach will facilitate drug development and advance precision medicine for PLEC.
